# Examining the effectiveness of a training program on improving knowledge, functional skills, and attitude in natural disaster volunteers

**DOI:** 10.3389/fpubh.2024.1321535

**Published:** 2024-04-24

**Authors:** Fereshteh Amini, Alireza Hidarnia, Fazlollah Ghofranipour, Mohammad Esmaiel Motlagh

**Affiliations:** ^1^Faculty of Medical Sciences, Tarbiat Modares University and Educational Board of Department of Internal Surgery, Faculty of Nursing and Midwifery, Tehran University of Medical Sciences, Tehran, Iran; ^2^Department of Health Education and Health Promotion, Faculty of Medical Sciences, Tarbiat Modares University, Tehran, Iran; ^3^Department of Community Medicine, School of Medicine, Ahvaz Jundishapur University of Medical Sciences, Ahvaz, Iran

**Keywords:** knowledge, functional skills, attitude, training program, volunteers, disasters

## Abstract

**Introduction:**

Disaster relief volunteers must have certain psychological and cognitive characteristics. Therefore, the present study aimed to investigate the effectiveness of the training program on improving knowledge, functional skills, and attitude among disaster volunteers.

**Methods:**

A quasi-experimental study was conducted in 2023 in Iran, including an intervention and control group and follow-ups. Ninety health volunteers were randomly selected and divided into two groups of 45. The experimental group received the training program for an hour a week for three months. The control group received no intervention. The participants of both groups completed the disaster preparedness questionnaire at the pre-test, post-test, and one and three months after the intervention. Data was analyzed using SPSS “version 26” software in the methods section.

**Results:**

The intervention effect were significant in increasing the volunteers’ functional skills (*F* = 3.75), knowledge (*F* = 166.50), and attitude (*F* = 1.74), all in (*p* = 0.001). According to the results, this effect was stable over time for functional skills (*F* = 1.83) and knowledge (*F* = 18.04) all in (*p* < 0.05).

**Conclusion:**

Training programs can help improve skills, attitudes and knowledge in natural disaster volunteers. Researchers interested in the field of natural disaster relief, particularly health researchers, could consider further examining the aforementioned topics in their studies.

## 1 Introduction

Natural disasters include earthquakes, droughts, floods, hurricanes, blizzards, and so on. Disaster preparedness is the ability of public health and healthcare systems, communities, and individuals to anticipate, protect, rapidly respond, and recover when coping with disasters (1). In other words, disaster preparedness is an integrated process that involves a variety of activities and resources as opposed to separate tasks. This necessitates readiness across various domains, ranging from education and logistics to healthcare (2). The World Health Organization’s (WHO) health policy guidelines emphasize the importance of training and professional development of individuals involved in disaster management (3). Indeed, in critical situations such as natural disasters, material, physical, and mental resources are weakened, and the needs of vulnerable people are not met. Vulnerable people do not have access to standard resources employed to prepare them for coping with, responding to, and recovering from disasters (4). In such circumstances, volunteers are valuable people who play key roles by improving people’s experiences, creating strong connections between the community and services, facilitating care integration, promoting public health, and reducing health inequities (5).

Volunteers deal with individuals’ personal, family, social, health, and psychological problems in disasters and similar situations (6). Since volunteers are considered role models in the society, they can have long-term effects on people’s perceptions, beliefs, and attitudes (7). Having functional skills can help volunteers improve their performance during disasters (8). These skills are related to personal capacity, level of knowledge, and inner satisfaction (9). With the advancement of technology and science, volunteers can now easily access new knowledge in various health fields (10). However, knowledge alone is not enough and volunteers need skills and attitude to manage crises (11). Broadly speaking, volunteers’ knowledge, skills, and attitudes can comprise ethics, emergency maneuvers, personal protective equipment, public health measures, awareness of specific disasters, incident command system (ICS), disaster triage, and emergency planning (12).

Continuous volunteer training is essential for their professional growth and can help them acquire new skills, knowledge, and competencies, which are necessary for the tasks they undertake (13). It can increase their confidence, performance, and effectiveness in providing health services (14). Studies have shown that trained volunteers are more likely to have a positive impact on victims health status (15). Health volunteer training should be designed to meet the specific needs of their roles and responsibilities, providing the necessary knowledge and skills to perform their duties effectively (16, 17). Education and training should be ongoing to ensure that volunteers stay up-to-date with the latest trends, practices, and policies in health care (18). Several studies have pointed out the role of education in increasing people’s safety during disasters and emergencies (19). Continuous education should be provided to nurses to improve their preparedness and motivation at times of crisis (20). Implementing training programs as part of the disaster management cycle improves volunteer preparedness (21). In other words, it will raise awareness among health staff regarding available programs to deal with disasters, increase their participation in planning and solving problems, and improve their skills to perform their responsibilities during disasters (22). A study conducted in the United States indicates that training volunteers enhances their management in different dimensions (23). Another study showed that evidence-based training enhances emergency preparedness skills in volunteers (24). A systematic review indicated that training programs are effective in increasing volunteers’ knowledge (12).

To our knowledge, there is no study that has examined the impact of an educational program on improving functional skills, knowledge, and attitude among natural disaster volunteers especially in Iran. In this country, we face many natural disasters such as floods, earthquakes, and so on. Therefore, we need training programs to increase the knowledge, attitude and skills of the volunteers. In Iran, natural disaster volunteers enroll in the Red Crescent Organization to be able to participate in volunteering activities, and are often ready to receive training in this field. For this reason, the innovative aspect of this study was in the design of the training program and its validation. Therefore, the present study aimed to investigate the effectiveness of the training program in improving their knowledge, skills, and attitude in natural disasters in Iran.

## 2 Methods

As the hypothesis of this study seeks to investigate the effectiveness of the training program in strengthening knowledge, functional skills and attitude in disaster volunteers, therefore, a quasi-experimental study was conducted in 2023 in Iran, including an intervention and a control group and follow-ups. Using Cohen’s formula (1988) and considering the first and second type errors, and the expected average difference in the study groups, the minimum sample size was estimated at 15. Nevertheless, 90 health volunteers were randomly selected and assigned to two groups of 45. The main researcher communicated with the volunteers after attending the Red Crescent Office and becoming a member of their social networks. Once she explained the research objectives she asked the volunteers to contact her. The inclusion criteria were, willingness to participate, having at least one year of volunteering experience, being physically fit for voluntary service delivery, and being aged 25–40 years old. Exclusion criteria included, missing more than two sessions of the educational program and being physically unfit for health and medical service delivery. The intervention group received the ‘Knowledge, Functional Skills, and Attitudes Training Program’ designed by the main researcher for an hour a week for three months. The control group received no intervention. The participants of both groups completed the disaster preparedness questionnaire by Ghanbari et al. (25) at the pre-test, post-test, and one and three months after the intervention at the follow-ups.

### 2.1 Ethical considerations

**Figure 1:**
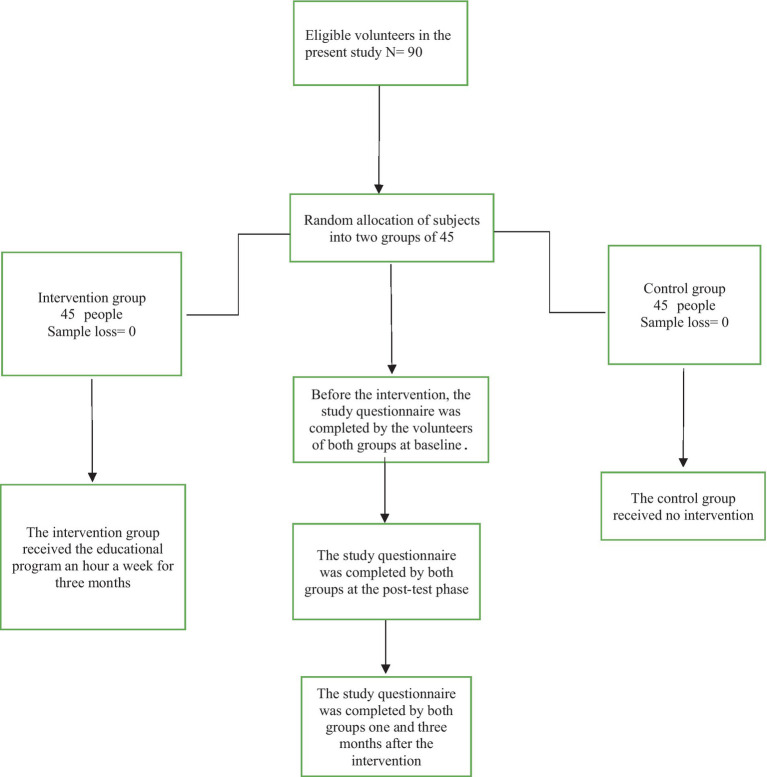
Consort flowchart of the present study.

The purpose of this study was explained to the study group at the beginning of the study and volunteers were ensured that their names and demographic characteristics would remain confidential. Furthermore, the intervention group was told that they could leave the study whenever they wanted. Written informed consent was taken from the participants after the aforementioned were explained to the participants (see Figure 1).

### 2.2 Tools

A demographic questionnaire was designed, including age, gender, marital and educational status, and professional experience.

The other tool used was *Ghanbari et al.’s Natural Disaster Preparedness Questionnaire* (2010). This questionnaire contains 72 items that are scored as Yes (1) and No (0). Therefore, the range of scores is between 0 and 72. This questionnaire has three subscales; attitude, functional skills, and knowledge. The content validity of this questionnaire has been approved by experts. Cronbach’s alpha coefficient was calculated for the subscale of knowledge at 0.61, attitude at 0.88, functional skills at 0.89, and 0.87 for the whole questionnaire (25). Here, Cronbach’s alpha was calculated at 0.89 for the entire questionnaire.

*The Knowledge, Functional Skills, and Attitudes Educational Program (Training Program)* The Knowledge, Functional Skills, and Attitudes Training Program was developed by the main researcher of this study, and content validity was approved by the health experts. This program was held an hour a week for three months for the intervention group (12 sessions) (Table 1).

**Table 1:** Contents and sessions of the training program.

Axis	Contents	Sessions
Functional skills	Introducing the program and the volunteers to one another and identifying natural disasters in Iran	One
	Theoretical and practical aspects of emergency management	Two
	Dealing with real and potential risks in different situations	Three
	Crisis management skills	Four
	Coping skills during stress and tension and psychological health management	Five
Attitude	Desire, motivation, and emotional management during disasters	Six
	Being mentally prepared for these conditions	Seven
	Cognitive and psychological flexibility in the face of natural disasters	
	Cognitive empowerment	Eight
Knowledge	Passing on correct and accurate information to people at critical times	Nine
	Sharing information with other volunteers during a crisis	Ten
	How to put knowledge into action and how to expand it through skills	Eleven
	Summary of sessions, and farewell	Twelve

For inferential analysis, after checking the underlying assumptions, analysis of variance of repeated measurements was used. After collecting the data, it was analyzed descriptively and inferentially using SPSS statistics version 26.

This study was registered in the Iranian Registry of Clinical Trials under registration number IRCT20180712040443N1.

## 3 Results

The number of males and females in the intervention group were 20 and 27, respectively, and in the control group they were 18 and 27, respectively. The majority of the participants in both the intervention and control groups held bachelor degrees. The volunteers’ age range and duration of professional experience are illustrated in Table 2.

**Table 2:** Demographic characteristics of the subjects.

Demographic characteristics			Control intervention	Control intervention
Gender	Male	Number	20	18
		Percent	44.44	40
	Female	Number	27	27
		Percent	60	60
Education level	Diploma	Number	9	3
		Percent	20	6.7
	Bachelor	Number	22	27
		Percent	48.9	60
	Master and higher	Number	14	15
		Percent	31.1	33.3
Age range	<25	Number	19	15
		Percent		
	25–30	Number	12	12
		Percent		
	30<	Number	20	12
		Percent		
Work experience	<2	Number	16	18
		Percent		
	2–4	Number	13	16
		Percent		
	4–6	Number	6	9
		Percent		
	6<	Number	8	4
		Percent		

The results of the Shapiro–Wilk test showed the normal distribution of the research variables. The confirmation of the null hypothesis in the study’s variables affirms the assumption of equal variances. This confirmation is observed in the variables of knowledge (*p* = 0.449, *F* = 0.584), attitude (*p* = 0.594, *F* = 0.289), and functional skills (*p* = 0.094, *F* = 2.904). Therefore, the assumptions were confirmed to implement a mixed-model analysis of variance (Table 3).

**Table 3:** The results of the Shapiro–Wilk and Levin tests in the examined variables.

Variable	Shapiro–Wilk		Levin test	
	*Z*	Significance level	*F*	Significance level
Knowledge	0.984	0.988	0.584	0.449
Attitude	0.946	0.470	0.289	0.594
Functional skills	0.935	0.327	2.904	0.094

Table 4 shows the adjusted means of knowledge, attitude, and functional skills of the disaster volunteers before and after the intervention and follow-ups in the studied groups. There was a significant increase in the mean knowledge, functional skills, and attitude of the intervention group compared to the control group after the intervention.

**Table 4:** The averages of the information, motivation, and skill level of health volunteers before and after the educational intervention.

Group	Pre-test	Adjusted average	Standard error	95% Confidence interval	
				Lower limit	Upper limit
Information					
Intervention	Pre-test	13.867	0.469	12.935	14.798
	Post-test	25.200	0.315	24.574	25.826
	1-month follow up	25.133	0.360	24.418	25.849
	3-month follow up	23.667	0.395	22.881	24.452
Control	Pre-test	12.022	0.469	11.091	12.954
	Post-test	12.511	0.315	11.886	13.137
	1-month follow up	12.356	0.360	11.640	13.071
	3-month follow up	12.622	0.395	11.837	13.408
Motivation					
Intervention	Pre-test	72.467	1.090	70.300	74.634
	Post-test	75.778	0.707	74.373	77.183
	1-month follow up	73.489	0.866	71.767	75.211
	3-month follow up	74.733	1.012	72.723	76.744
Control	Pre-test	67.800	1.090	65.633	69.967
	Post-test	67.511	0.707	66.106	68.916
	1-month follow up	67.733	0.866	66.0121	69.455
	3-month follow up	68.333	1.012	66.323	70.344
Skill					
Intervention	Pre-test	10.800	0.998	8.817	12.783
	Post-test	14.422	1.092	12.252	16.592
	1-month follow up	15.644	1.023	13.611	17.678
	3-month follow up	16.978	1.040	14.911	19.045
Control	Pre-test	8.489	0.998	6.506	10.472
	Post-test	8.822	1.092	6.653	10.992
	1-month follow up	8.778	1.023	6.744	10.811
	3-month follow up	9.00	1.040	6.933	11.067

Based on the two-factor variance test, the time factor (*p* = 0.001, *F* = 196.250), and the intervention’s effect on the time factor (p = 0.001, *F* = 166.506) significantly increased the volunteers’ mean knowledge (Table 5), and so did the group factor (*F* = 512.429, p = 0.001). However, the effect size of the group factor was equal to 0.85, thus, the 85 percent variance increase may be attributed to this intervention. In simpler terms, 85 percent of volunteers in the intervention group experienced a rise in their mean knowledge level when compared to the control group.

**Table 5:** The results of two-factor variance analysis of information level in the intervention and control groups.

Status	Total squares	df	Mean square	*F*	*p*-value	Eta
Information						
Time factor	2171.233	3	723.744	196.250	0.001**	0.69
Intervention (time × intervention)	1842.167	3	614.056	166.506	0.001**	0.65
Group factor	8275.211	1	8275.211	512.429	0.001**	0.85
Motivation						
Time factor	144.053	3	48.018	1.633	0.182	0.018
Intervention (time × intervention)	153.875	3	51.292	1.745	0.158	0.019
Group factor	3540.669	1	3540.669	52.362	0.001**	0.37
Skill						
Time factor	551.344	3	183.781	5.102	0.002**	0.06
Intervention (time × intervention)	405.978	3	135.326	3.757	0.011*	0.04
Group factor	2912.711	1	2912.711	33.798	0.001**	0.28

**Significance level at 0.01.

*Significance level at 0.05.

The time factor (*p* = 0.182, *F* = 1.633), and the intervention effect on the time factor (*p* = 0.158, *F* = 1.745) were not significant in increasing the attitude level of the volunteers. Moreover, the group factor (*p* = 0.001, *F* = 52.362) significantly increased the attitude level of the volunteers. In other words, although the time factor and intervention effect on the time factor increased the mean attitude level of the intervention group, however, this increase was not statistically significant. The effect size of the group factor was equal to 0.37 (Eta = 0.37); so, overall, 37 percent of the volunteers in the intervention group experienced an increase in their mean attitude scores compared to the control group.

The time effect (*F* = 5.102, *p* = 0.002) and the intervention effect (*F* = 3.757, *p* = 0.001) were significant in increasing the volunteers’ functional skills. Also, the group factor (*F* = 33.798, p = 0.001) significantly raised the mean level of functional skills. In other words, the intervention improved the volunteers’ functional skills. Furthermore, the effect size of the group factor stood at 28 percent, meaning the educational intervention accounted for 28 percent of the variance in improved functional skills among the health volunteers in the intervention group (Table 6).

**Table 6:** Bonferroni test results to compare the functional skills and information level of health volunteers in the pre-test, post-test, and follow-ups.

Group	Test stages	Mean difference	Standard error	Significance level	95% Confidence interval	
					Lower limit	Upper limit
Functional skills						
Pre-test	Pre-test	−2.978	0.877	0.014*	−4.345	−0.389
	1-month follow up	2.567*	0.843	0.018*	−4.843	−0.291
	3-month follow up	−3.344	0.803	0.001**	−5.511	−1.178
Control	Pre-test	1.978	0.877	0.160	−0.389	4.345
	1-month follow up	−0.589	0.914	0.999	−3.056	1.878
	3-month follow up	−1.367	1.038	0.999	−4.168	1.435
3-month follow up	Pre-test	2.567*	0.843	0.018*	0.291	4.843
	Post-test	0.589	0.914	0.999	−1.878	1.584
	3-month follow up	−0.778	0.875	0.999	−3.140	1.584
1-month follow up	Pre-test	3.344*	0.803	0.001**	1.178	5.511
	Post-test	1.367	1.038	0.999	1.435	4.168
	1-month follow up	0.778	0.875	0.999	−1.584	3.140
Information						
Pre-test	Post-test	−5.911	0.237	0.001**	−6.551	−5.271
	1-month follow up	−5.800	0.343	0.001**	−6.725	−4.875
	3-month follow up	−5.200	0.334	0.001**	−6.102	−4.298
Post-test	Pre-test	5.911	0.237	0.001**	5.271	6.551
	1-month follow up	0.111	0.242	0.999	−0.541	0.764
	3-month follow up	0.711	0.294	0.106	−0.083	1.505
1-month follow up	Pre-test	5.800	0.343	0.001**	4.875	6.725
	Post-test	−0.111	0.242	0.999	−0.764	0.541
	3-month follow up	0.600	0.248	0.105	−0.069	1.269
3-month follow up	Pre-test	5.200	0.334	0.001**	4.298	6.102
	Post-test	−0.711	0.294	0.106	−1.505	0.083
	1-month follow up	−0.600	0.248	0.105	−1.269	0.069

** Significant level at 0.01.

*Significant level at 0.05.

There were significant differences between the functional skills (*F* = 1.83), and knowledge (*F* = 18.04) scores at pre-test and post-test stages at one-month and three-month follow-ups. However, there was no significant difference between the one-month and three-month follow-ups in attitudes (*F* = 0.03) (*p* > 0.05). So, the intervention effect was observed in the time series.

## 4 Discussion

The purpose of the present study was to investigate the effectiveness of the training program in improving knowledge, skills, and attitudes in natural disaster volunteers. Based on our results, the training program enhanced their attitude, functional skills, and knowledge at the post-test stages. However, only improvements in functional skills and knowledge sustained at the one-month, and three-month follow-ups.

Our study’s results are consistent with those of Iizuka (23), Levina Chandra and Chan (24), Sena et al. (21), and Ahmadi Marzaleh et al. (12). In all these studies the training program was effective in improving the volunteers’ skills, knowledge, and attitude. In explaining this finding, it seems that the lack of trained volunteers in disasters is a big challenge for healthcare systems worldwide, especially in low-income countries. As studies indicate, preparedness comes with higher knowledge and skills, which in turn help to cope with disasters (26). Trained volunteers can prove beneficial by transferring knowledge and experiences to local communities and improving public awareness concerning the management of various crises or disasters (27). Raising awareness and knowledge among volunteers improves their attitudes at times of crisis too (28).

Here, the training program improved the volunteers’ functional skills, creating a friendly atmosphere and improving their communication skills at the post-tests and later follow-ups. However, it is crucial to focus on the appeal of educational subjects and packages to encourage volunteers to sustain their voluntary contributions. Moreover, functional skills need to be regularly practiced and kept up-to-date through continuous educational programs. Knowledge gained during regular educational sessions increases volunteers’ competence (e.g., communication skills) and self-confidence and may encourage them to continue volunteering due to their enhanced attitude (29). Therefore, there is a mutual interaction between knowledge and attitude in individuals opting for voluntary participation and promotion. Over time, knowledge is stable, but attitude can easily change. From a cognitive viewpoint, attitude is mainly based on a person’s behavior rather than cognition or feeling. Perhaps, this is because attitude has emotional characteristics and is influenced by changes in one’s mentality (30). Thus, attitude is influenced by one’s emotions (31). Volunteers assisting in disaster events may adjust their attitudes based on various factors, including their personal circumstances and demographic characteristics like age and change in socio-economic status. Ongoing support provided to volunteers plays a key role in helping them overcome negative expectations and emotions, as attitudes significantly influence our expectations, emotions, affects, and motivation (32, 33). Among the limitations of this study were the lack of cooperation on behalf of some of the volunteers under study, and coordinating the sessions with them.

## 5 Conclusion

Based on our results, the training program was able to improve functional skills, knowledge, and attitude among disaster volunteers, and this effect was stable over time for functional skills and knowledge. Therefore, training programs that address skills, attitudes, and knowledge can be taken into consideration for empowering natural disaster preparedness among volunteers. Researchers and particularly health researchers looking to investigate in these fields should consider conducting interventions on smaller sample sizes to control the effect of the group size.

## Data availability statement

The original contributions presented in the study are included in the article/supplementary material, further inquiries can be directed to the corresponding author.

## Ethics statement

The studies involving humans were approved by Tarbiat Modares University (Research Ethics Committee). The studies were conducted in accordance with the local legislation and institutional requirements. The participants provided their written informed consent to participate in this study.

## Author contributions

FA: Data curation, Investigation, Methodology, Writing – original draft, Writing – review & editing. AH: Conceptualization, Formal analysis, Investigation, Resources, Supervision, Writing – original draft, Writing – review & editing. FG: Conceptualization, Supervision, Writing – original draft, Writing – review & editing. MM: Supervision, Writing – review & editing.
